# The downregulation of ΔNp63 in p53-deficient mouse epidermal tumors favors metastatic behavior

**DOI:** 10.18632/oncotarget.4353

**Published:** 2015-07-01

**Authors:** Olga Bornachea, Fernando F. López-Calderón, Marta Dueñas, Carmen Segrelles, Corina Lorz, Cristian Suárez-Cabrera, María Marañón, Beatriz Paradela-Dobarro, Mirentxu Santos, Jesús M. Paramio

**Affiliations:** ^1^ Molecular Oncology Unit, CIEMAT, 28040 Madrid, Spain; ^2^ Molecular Oncology, Institute of Biomedical Investigation University Hospital, 28041 Madrid, Spain

**Keywords:** p63, p53, metastasis, miRNA, skin

## Abstract

The TP63 gene codes for two major isoform types, TAp63 and ΔNp63, with probable opposite roles in tumorigenesis. The ΔNp63α protein is frequently amplified and overexpressed in different epithelial tumors. Accordingly, it has been considered a potential oncogene. Nonetheless, a possible metastatic suppressor activity has also been suggested based on the experimental observation that its expression is reduced or even absent in advanced invasive tumors. Such metastatic suppressor activities are often related to tumors bearing point mutated *TP53* gene. However, its potential roles in TP53-deficient tumors are poorly characterized. Here we show that in spontaneous tumors, induced by the epidermal-specific *Trp53* ablation, the reduction of ΔNp63 expression is an early event, whereas it is re-expressed in the lung metastatic lesions. Using knock down and ectopic expression approaches, we show that ΔNp63 expression opposes the epithelial-mesenchymal transition and reduces the metastatic potential of the cells. This process occurs through the modulation of ΔNp63-dependent downstream targets (including transcription factors and microRNAs) likely to play metastatic roles. Further, ΔNp63 also favors the expression of factors involved in iPS reprogramming, thus suggesting that it can also modulate specific stem cell traits in mouse epidermal tumor cells. Overall, our data assign antimetastatic roles to ΔNp63 in the context of p53 deficiency and epidermis.

## INTRODUCTION

The *TP63* gene forms, together with *TP53* and *TP73*, the so-called p53 family. These genes produce multiple isoforms due to alternative splicing and alternative promoter usage [1]. The resulting proteins exert specific roles in development, differentiation and cancer progression [2]. This last aspect is highlighted by the extremely frequent mutations affecting the *TP53* gene in multiple human tumors, which in general are associated with increased malignancy and poor clinical outcome [3–5]. However, the roles of the other p53 family members in the context of cancer are less understood. This could be attributed to the differential expression of multiple isoforms with distinct properties. In the case of *TP63*, by the use of alternative promoters, two major isoform types are transcribed, named TAp63 and ΔNp63, with different and often opposing functional properties [6]. TAp63 can induce cell cycle arrest and apoptosis and participates in the DNA damage response [7, 8], functions commonly attributed to the p53 family. ΔNp63 is primarily expressed in the basal layer of most stratified epithelia and exerts essential functions in epithelial development and differentiation [9, 10]. In the context of cancer, the roles of these isoforms and their possible crosstalk with p53 are still partially obscure. With few exceptions, mutations of the *TP63* gene are rare in human malignancies, suggesting that it is not a canonical tumor suppressor. On the contrary, several human tumors display overexpression and also amplification of this gene, suggesting a potential oncogenic role [11]. This last aspect is particularly relevant for the ΔNp63α isoform, which is specifically amplified and overexpressed in multiple stratified epithelia-derived tumors [12], promotes the activation of stratified-epithelia relevant oncogenic pathways [13], can mediate resistance to chemotherapy [14], and overcome the oncogene-induced senescence [15]. Nonetheless, in some aggressive metastatic epithelial tumors the expression of ΔNp63 is reduced and often lost, suggesting potential roles as metastasis suppressor [16–21]. This is in agreement with the observed ability of ΔNp63 to bind and modulate the expression of a variety of genes, including transcription factors, adhesion and signaling molecules, and also several miRNAs [14, 20, 22–25]. In addition, these roles may also explain why the limited metastatic spreading of spontaneous tumors arising in *Trp53*−/− mice is significantly enhanced by simultaneous ablation of the *Trp63* gene [26]. However, the potential opposite differences affecting the functional roles of TAp63 and ΔNp63 isoform types makes the possible contribution of these proteins a complex issue. Therefore, further research is clearly needed to ascertain the actual oncogenic and/or metastatic suppressor roles of ΔNp63 in order to consider possible targeted therapies.

The functional interaction between p53- and p63-dependent signaling pathways has remained poorly investigated until recently, when two new mechanisms have been described. These implicated the physical interaction between p63 and mutant p53 in the context of TGFβ signaling, leading to the inhibition of various genes involved in metastasis and whose expression is regulated by p63 [27, 28]. Although the possible differences affecting TAp63 and ΔNp63 isoform types in this context have not been completely elucidated, these findings reinforce a possible role of p63 as a metastasis suppressor and also may help to explain the gain of function of missense mutations of the *TP53* gene and their involvement in the increased malignancy observed in human p53-mutant tumors [29–31]. Nevertheless, human tumors also frequently display absence of p53 expression due to deletion of the *TP53* gene, and the occurrence of *TP53* gene point mutations leading to premature stop codon generation. The potential roles of p63 in this context of p53 loss are almost completely unknown.

We have previously reported that the specific ablation of *Trp53* in mouse stratified epithelia (hereafter *Trp53^ΔEpi^* mice) leads to spontaneous tumor development [32]. These tumors arise primarily in the epidermis and their onset is accelerated by the concomitant loss of other tumor suppressors such as *Rb1* and *Pten* [32, 33]. Importantly, the transcriptome analyses of these mouse tumors revealed massive overlapping with multiple human tumors characterized by poor prognosis, metastatic spreading and p53 mutation [34]. This overlap is not limited to human skin cancers, and includes multiple tumors of high clinical relevance arising in tissues such as breast and lung [35]. Interestingly, the transcriptome analysis also revealed a significant enrichment of stem cell-like signatures in these p53-deficent tumors [34], which are also associated with the metastatic spreading of human tumors [36]. More recently we also demonstrated the high metastatic capacity of these *Trp53^ΔEpi^* tumors, and we characterized a role for specific miRNAs in this process through a possible modulation of the epithelial mesenchymal transition (EMT) signaling pathway [37]. EMT is a critical process during embryonic development and has also been recognized as a potential mechanism for carcinoma metastasis [38, 39]. During EMT, epithelial cells lose cell-to-cell adhesion and cell polarity to gain mesenchymal features, providing motility and invasiveness. This is accomplished by an intricate network of transcription factor activation, including TWIST, SNAIL, SLUG, ZEB1 and ZEB2 which are also under epigenetic regulation through various miRNAs [38, 40, 41]. Importantly, recent evidences also support a role for ΔNp63 modulating EMT [18, 19]. Here we report that spontaneous tumors arising in *Trp53^ΔEpi^* mice display early repression of ΔNp63 expression. Using overexpression and knock down approaches, we also observe that altered expression of ΔNp63 leads to the disturbed expression of various transcription factors and miRNAs involved in the acquisition of stem cell-like characteristics and in the EMT process.

## RESULTS

### Reduced ΔNp63 expression is an early event in *Trp53^ΔEpi^* spontaneous tumors

**Figure 1:**
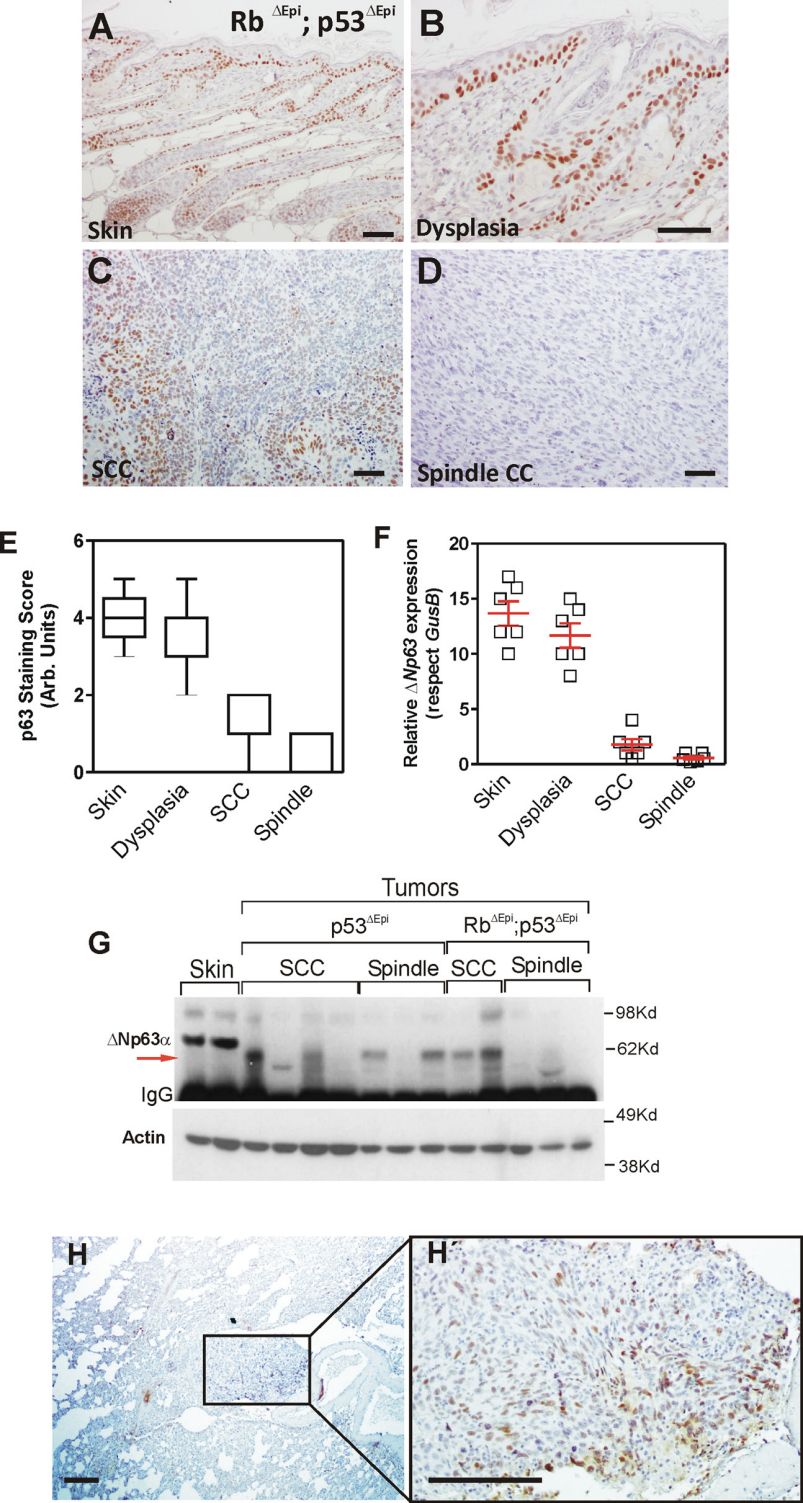
ΔNp63 downregulation is an early event in spontaneous *Trp53*-deficient epidermal tumors **A–D.** Representative examples of p63 expression in non lesional epidermis (A), dysplasia (B), moderately differentiated tumor (C) and undifferentiated tumor from *Rb1^ΔEpi^*;*Trp53^ΔEpi^* mice as assessed by immunohistochemistry. **E–F.** Summary of p63 expression in samples determined by immunohistochemistry (E) or qRT-PCR (F) **G.** Immunoblot of the quoted protein extracts showing the expression of p63 isoforms. Note the lack of ΔNp63α expression in tumors, regardless their genotype, and the presence of a fast migrating isoform in some of them (denoted by red arrow). **H, H'.** Representative examples of p63 expression in lung metastases. H' is a high magnification of the area denoted in H covering the metastatic nodule. Bars = 150 μm

Immunohistochemical analyses revealed that the pattern of p63 expression in the skin of *Trp53^ΔEpi^* and *Trp53^ΔEpi^*;Rb1*^ΔEpi^* mice is undistinguishable from control wt mice (Fig. 1A, 1E), and remains expressed, mainly in the basal layer, in areas of epidermal dysplasia (Fig. 1B, 1E). However, its expression is drastically reduced in squamous cell carcinoma (SCC) (Fig. 1C, 1E) and almost completely absent in spindle cell carcinomas (SpCC) (Fig. 1D, 1E). Since the antibody used reacts with all p63 isoforms, we also performed a qRT-PCR analysis using primers specific for the ΔNp63 isoform. This revealed the systematic reduction of this isoform in mouse tumors (Fig. 1F). These findings were corroborated by western blot analyses (Fig. 1G), which also showed that ΔNp63α, the major isoform expressed in skin, is absent in most tumors, although some of them expressed p63 isoforms of lower molecular weight (indicated by arrow in Fig. 1G). We also studied the p63 expression in the spontaneous metastatic lesions arising in the lungs of *Trp53^ΔEpi^* or *Trp53^ΔEpi^*;Rb1*^ΔEpi^* mice. We found that these lesions display positive p63 staining (Fig. 1H, H'), suggesting that the observed reduction of ΔNp63α expression in the primary tumors is not attributable to gene loss or deletion, but rather to a transcriptional repression.

### The expression of ΔNp63 opposes EMT in *Trp53^ΔEpi^* tumors

**Figure 2:**
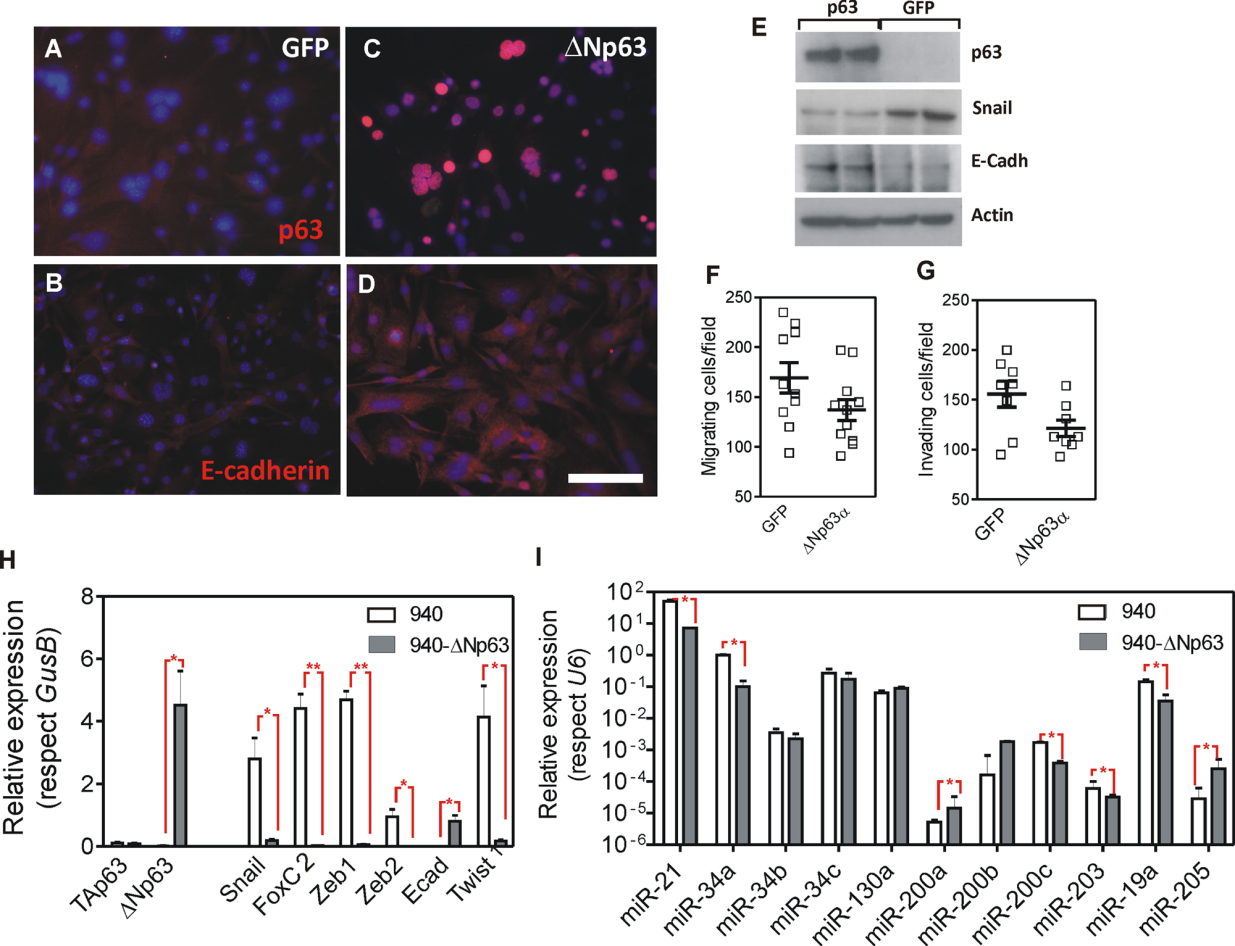
Expression of huΔNp63α in 940 cells ameliorates EMT process **A–D.** Representative immunofluorescence images of 940 cells traduced with GFP (A, B) or ΔNp63α (C, D) showing the expression and localization of p63 (A, C) and E-Cadherin (B, D). **E.** Immunoblot of the quoted protein extracts showing the expression of p63, Snail and E-cadherin. Actin was used to normalize loading. **F, G.** Determination of migratory (F) and invasive (G) properties of 940 cells upon transduction with GFP or ΔNp63α coding lentiviruses. **H, I.** Determination of the expression of transcription factors (H) and miRNAs (I) involved in EMT processes as assessed by qRT-PCR analyses. Data came from at least quadruplicated experiments. *denotes *p* value < 0.05, **denotes *p* value < 0.01 Bars = 150 μm

To study the functional consequences of the reduced p63 expression, we sought to express huΔNp63α in the 940 SpCC cells [37]. These cells were derived from a *Trp53^ΔEpi^*;Rb1*^ΔEpi^* mouse spontaneous primary tumor, which also showed lung metastasis [37]. Compared with COCA immortalized keratinocytes [42], the 940 cells are characterized by a spindle morphology (Supplementary Fig. 1A, 1B), absence of p53 and TAp73α expression, severe reduction of ΔNp63α expression, and increased expression of ΔNp73α (Supplementary Fig. 1C). In these cells, the pharmacological treatment to inhibit specific pathways that can mediate p63 repression leads to partial increase in *ΔNp63* (Supplementary Fig. 1D). However, its levels never reached those observed in immortalized COCA keratinocytes (Supplementary Fig. 1D). Previous data also showed that 940 cells are highly metastatic when injected either subcutaneously or in the tail vein of immunocompromised mice [37], whereas COCA cells are not tumorigenic [42]. We used a lentiviral vector, which also expressed the EGFP protein, to ectopically express huΔNp63α, and transduced cells were sorted based on EGFP fluorescence. Compared to the control EGFP transduced cells (Fig. 2A, 2E), the majority of the 940 cells transduced with huΔNp63α showed the expression of the transduced gene (Fig. 2C, 2E). Importantly, although no major changes in cell morphology were detected upon huΔNp63α expression (not shown), we observed a partial increase in E-cadherin expression, which was not properly localized at the cell-cell contacts (Fig. 2B, 2D, 2E). Moreover, the expression of huΔNp63α produces a limited inhibition of the migratory (Fig. 2F) and invasive (Fig. 2G) characteristics of the 940 cells. The silencing of the *Cdh1* gene (coding for E-cadherin) is an early marker and a prerequisite for the EMT process. This event is promoted by the concerted action of various transcription factors including Snail, FoxC2, Zeb1, Zeb2 and Twist1, which are also induced early in the spontaneous tumors arising in *Trp53^ΔEpi^* mice [37]. We observed that the expression of huΔNp63α in 940 cells induced a significant decrease in the expression of all these transcription factors that promote EMT (Fig. 2E, 2H). Remarkably, none of these genes have been identified as bound by p63 [20, 22–25], suggesting that such reduction is not directly mediated by p63 binding to their respective promoters.

Several miRNA modulate the EMT process [40, 43, 44] and are regulated by p53 and/or p63 [14, 24, 45]. These include miR21, miR200 family, miR34 family, miR130a, miR203, miR19a and miR205. We thus analyzed whether the expression of these miRNAs was affected by the ectopic expression of HuΔNp63α in 940 cells. We observed a significant reduction in the expression of miR21, miR34a, miR200c, miR203 and miR19a, whereas miR200a, miR200b and miR205 displayed increased expression in HuΔNp63α-expressing 940 cells (Fig. 2I). Importantly, the reduction of miR21, miR19a and miR203, and the increased expression of miR200a, miR200b and miR205 are compatible with a possible reduction in the metastatic potential of 940 cells upon ectopic expression of huΔNp63α, and may explain the indirect regulation of EMT-mediating transcription factors described above.

Collectively, these data indicate that p63 expression opposes EMT, probably through the indirect modulation of the expression of various transcription factors due to altered miRNA expression, but it is unable to completely reverse this process, as shown by the absence of significant changes in cell morphology and E cadherin localization.

### ΔNp63 expression delays metastatic spreading

**Figure 3:**
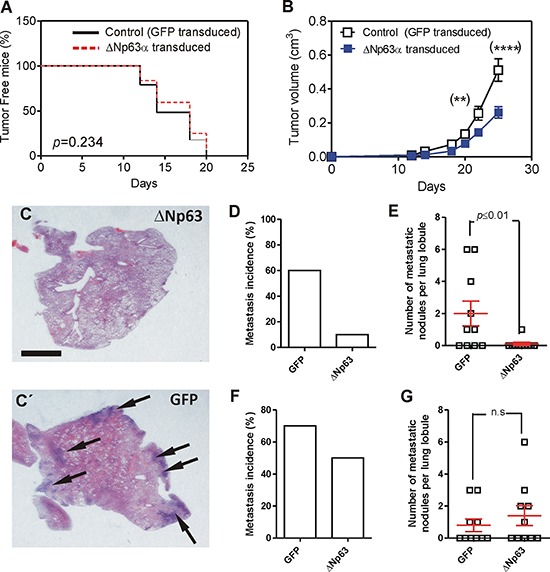
Expression of huΔNp63α decreases spontaneous metastasis development **A.** Tumor incidence upon subcutaneous injection of 940 cells upon transduction with GFP (black line) or ΔNp63α (red line) coding lentiviruses determined by Kaplan Meyer curves. *P* Value was determined by log rank test. **B.** Tumor growth of subcutaneously injected 940 (open squares) or huΔNp63α (blue squares) cells. *P* Value was determined by 2Way ANOVA followed by Bonferroni post-test. *denotes *p* value < 0.05, ****denotes *p* value < 0.0001. **C, C'.** Representative images of H&E stained lung lobule sections showing the presence of metastatic nodules (denoted by arrows) upon tumor induction by subcutaneous injection of 940 cells upon transduction with ΔNp63α (C) or GFP (C') coding lentiviruses. Bar = 0.5 cm **D, E.** Summary of lung metastasis incidence (D) and number of metastatic nodules per lung lobule (E) observed in mice bearing subcutaneously injected with 940 or huΔNp63α cells and sacrificed one month after injection. **F, G.** Summary of lung metastasis incidence (D) and number of metastatic nodules per lung lobule (E) observed in mice one month after surgical removing the subcutaneous tumors produced by 940 or huΔNp63α 940 cells.

To ascertain whether the functional consequences of the deregulated expression of miRNAs and reduced expression of transcription factors induced by huΔNp63α cells may result in altered metastatic spreading of these cells, we subcutaneously injected control or huΔNp63α-transduced cells in the flank of athymic (*nu/nu*) mice. We observed that mice developed tumors with similar incidence regardless the cells injected (Fig. 3A). Further, the spindle histology of the tumors was similar between control and huΔNp63α-transduced cells (not shown). However, the tumors generated by huΔNp63α-transduced cells displayed a partial delayed growth compared with the controls (Fig. 3B). In spite of this observation, the cell cycle profiles were similar in control and in huΔNp63α-transduced cells (Supp Fig. 2A).

**Figure 4:**
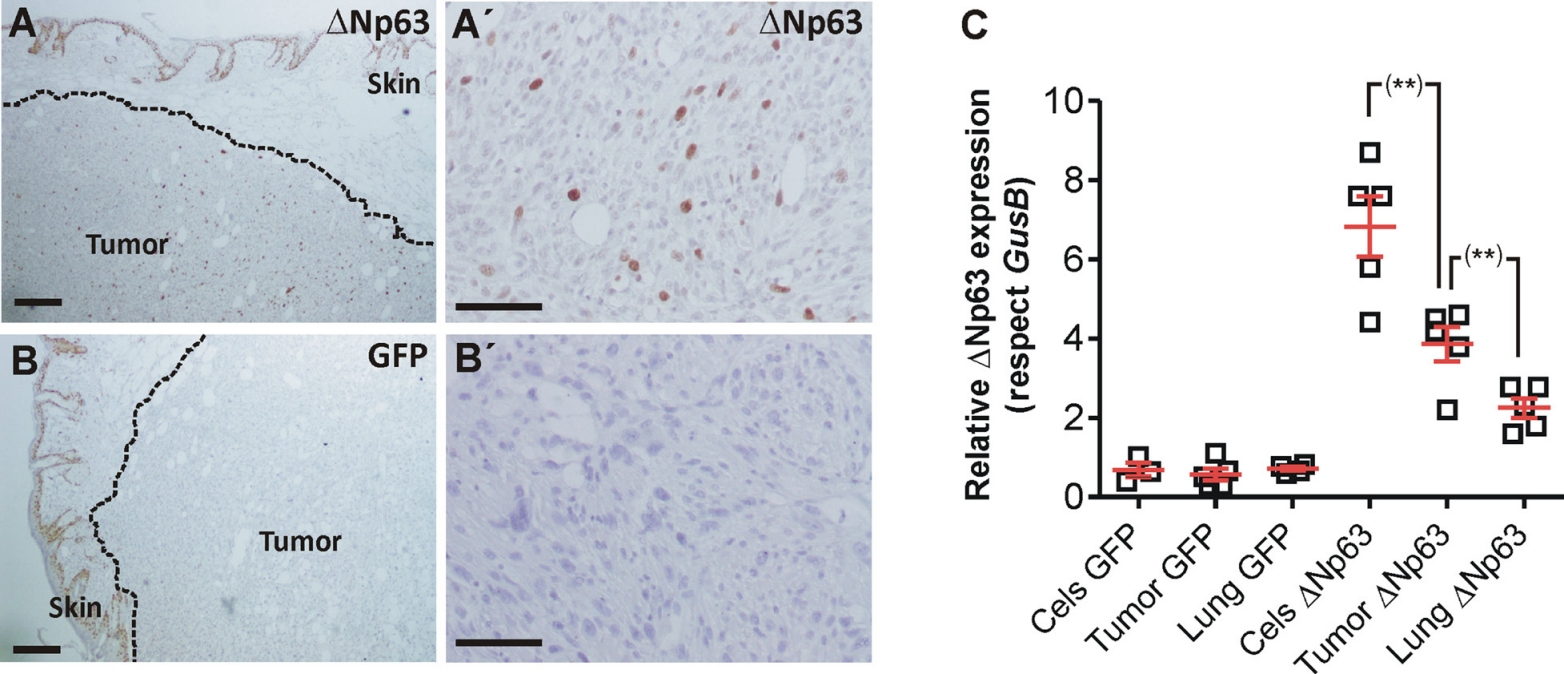
huΔNp63α expression is downregulated during spontaneous metastasis development **A, A'.** Representative immunohistochemistry image showing p63 expression in subcutaneous tumors produced by the injection of huΔNp63α 940 cells **B, B'.** Representative immunohistochemistry images showing p63 expression in subcutaneous tumors produced by the injection of 940 cells **C.** Summary of qRT-PCR determination of huΔNp63α expression upon transduction with GFP or ΔNp63α coding lentiviruses cells, and in the tumors and lung metastases after subcutaneous injection of the cells. Bars = 150 μm

To monitor possible metastatic outgrowths, the cohort of mice was split into two subgroups. In one of them the lungs were analyzed immediately after sacrifice, whereas in the other the subcutaneous tumors were surgically removed, and mice were euthanized one month later. In the first group the huΔNp63α-transduced displayed reduced lung metastasis incidence (percentage of lung lobules bearing metastatic lesions) compared to control cells (Fig. 3C, C', 3D), and also a reduced number of metastatic nodules (number of metastatic lesions per lung lobule) (Fig. 3C, C', 3E), indicating that the forced expression of huΔNp63α caused a delayed metastatic spreading. However, these differences were not observed when the tumors were surgically removed and mice kept alive for further 30 days (Fig. 3F, 3G). Nonetheless, we observed that the number of cells expressing huΔNp63α in the subcutaneous tumors was very low (Fig. 4A, A') compared with the parental cells injected (Fig. 2C). As expected, no p63 expression was observed in tumors produced by the injection of control EGFP-expressing cells (Fig. 4B, B'). To further support this finding, we performed qRT-PCR of huΔNp63α from transduced cells, subcutaneous tumors and lungs bearing metastatic nodules. We observed a significant reduction in tumors compared with cells, which is further reduced in the lungs (Fig. 4C).

**Figure 5:**
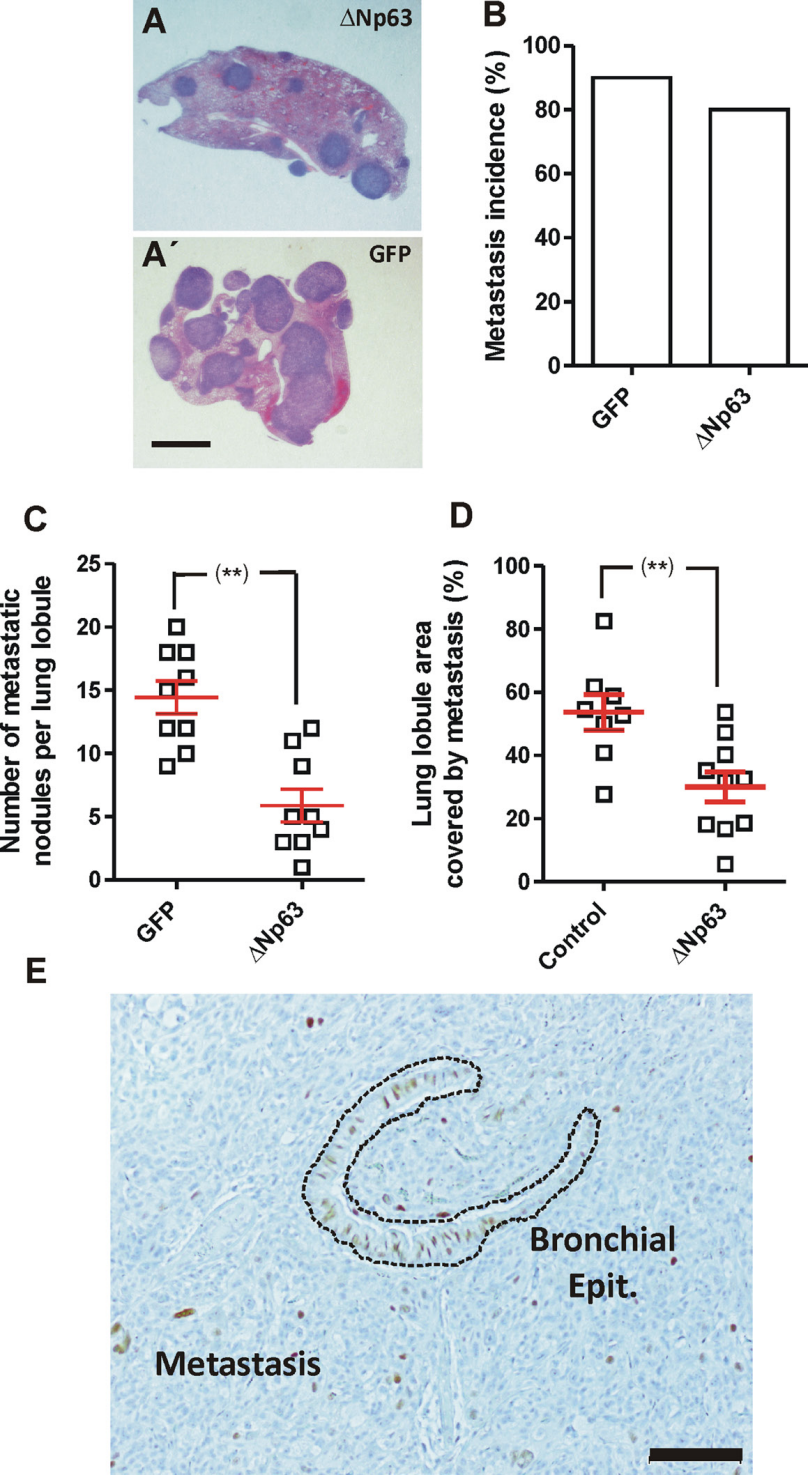
Expression of huΔNp63α decreases experimental metastasis development **A, A'.** Representative images of H&E stained lung lobule sections showing the presence of metastatic nodules upon tail vein injection of 940 cells upon transduction with ΔNp63α (A) or GFP (A') coding lentiviruses. Bar = 0.5 cm **B–D.** Summary of lung metastasis incidence (B), number of metastatic nodules per lung lobule (C) and lung area covered by metastatic nodules (D), observed in mice injected into the tail vein with 940 or huΔNp63α cells and sacrificed one month after injection. **denotes a *p* value < 0.01 **E.** Representative immunohistochemistry image showing p63 expression in a lung metastasis produced by the tail vein injection of huΔNp63α 940 cells. Bar = 250 μm.

The metastatic dissemination depends on various sequential events: invasion intravasation, survival in the blood stream, extravasation and colonization of the target organ. To study whether the last steps of the process were affected by huΔNp63α expression, control and huΔNp63α-transduced cells were injected in the tail vein of athymic (*nu/nu*) mice. Upon sacrifice we detected a reduced number of lung nodules in the case of huΔNp63α-transduced cells compared to control cells (Fig. 5A, A', 5C) in spite of similar metastatic incidence (Fig. 5B). The reduced lung colonization was further confirmed by measuring the lung area covered by nodules, which was also reduced in the case of huΔNp63α-transduced cells (Fig. 5D). Nonetheless, we also observed that the number of cells expressing huΔNp63α was severely low in the metastatic nodules (Fig. 5E), in agreement with the PCR observations (Fig. 4C). Collectively these findings indicated that huΔNp63α caused a partial inhibition of metastatic spreading. However, our data also suggested that huΔNp63α expression caused a potential growth disadvantage *in vivo*, and was probably silenced during tumor growth and metastatic spreading. This led to a reduced presence of cells expressing huΔNp63α in the tumors and metastases.

### Reduced ΔNp63 expression favors EMT

**Figure 6:**
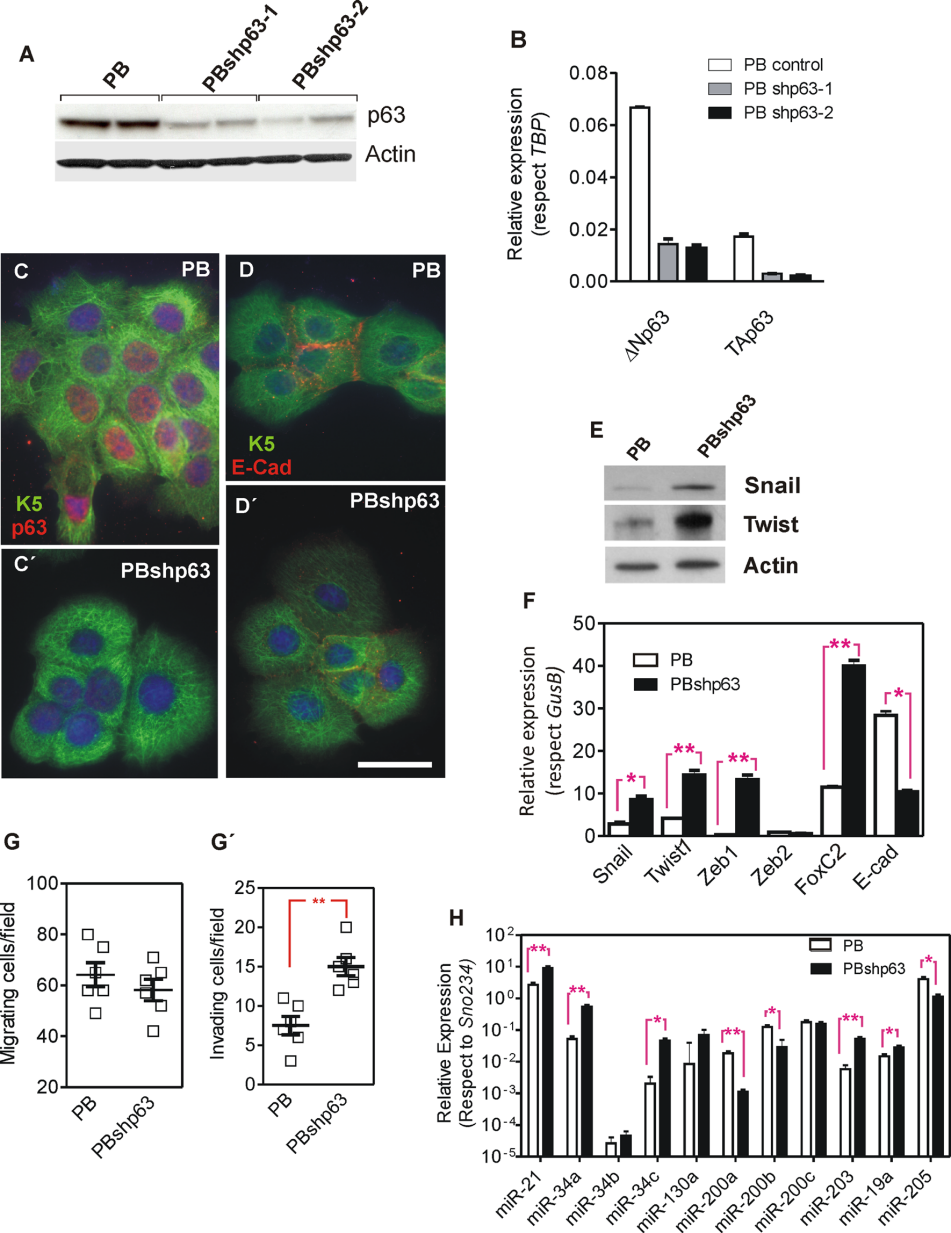
Knock down of p63 promotes a partial EMT process in PB transformed keratinocytes **A, B.** Determination of p63 expression by immunoblot (A) or qRT-PCR (B) in parental PB cells and PB cells transduced with lentiviral constructs coding for two different shRNAs against mouse *Trp63* gene. **C–D'** Representative immunofluorescence images of PB (C, D) and PBshp63 cells (C', D') showing in red the expression and localization of p63 (C, C') and E-Cadherin (D, D') and in green keratin K5. Bar = 20 μm. **E.** Immunoblot showing the expression of the quoted proteins in PB and PBshp63 cells. **F.** Determination of the expression of transcription factors involved in EMT processes, as assessed by qRT-PCR analyses, in PB and PBshp63 cells. **G G'.** Determination of migratory (G) and invasive (G') properties of PB and PBshp63 cells. **H.** Determination of the expression of miRNAs involved in EMT processes, as assessed by qRT-PCR analyses, in PB and PBshp63 cells. Data in F and H came from at least quadruplicated experiments. *denotes *p* value < 0.05, **denotes *p* value < 0.01

The above described results indicated that the forced expression of huΔNp63α in p53-deficient cells reduces metastatic behavior and the expression of genes and miRNAs that mediate EMT. To monitor whether the reduced expression of p63 facilitates these events, we used knock down approaches in PB keratinocytes. These cells were isolated from a chemically induced mouse papilloma and bear *Trp53* mutations [46]. Upon subcutaneous injection they render mostly differentiated SCCs and no metastasis [47]. We used two different shRNA lentiviral constructs that significantly reduced the endogenous p63 expression as confirmed by immunoblot (Fig. 6A) and qPCR (Fig. 6B) affecting both ΔN and TA isoforms. Importantly, although TAp63 can be detected by qPCR (Fig. 6B), its levels are extremely low (not shown), thus suggesting that the possible changes observed (see below) are not primarily attributable to the decreased TAp63 levels. Immunofluorescence analysis corroborated the reduction of ΔNp63 expression (Fig. 6C, C') and suggested a concomitant decrease in E-Cadherin in the p63 silenced cells (Fig. 6D, D'). Immunoblot and qPCR studies showed that the decreased ΔNp63 expression in PB cells was accompanied with a significant increase in Snail, Twist1, Zeb1 and FoxC2 expression, and confirmed the reduction of E-cadherin (Fig. 6E, 6F). In agreement, the reduced expression of p63 also accounted for increased invasiveness, but not migratory properties, of the PB keratinocytes (Fig. 6G, G'). Finally, the reduction of p63 levels also affected the expression of several miRNAs (Fig. 6H), in agreement with the data obtained with huΔNp63α-expressing 940 cells (Fig. 2I). These data indicated that reduced p63 expression favored EMT in PB keratinocytes.

**Figure 7:**
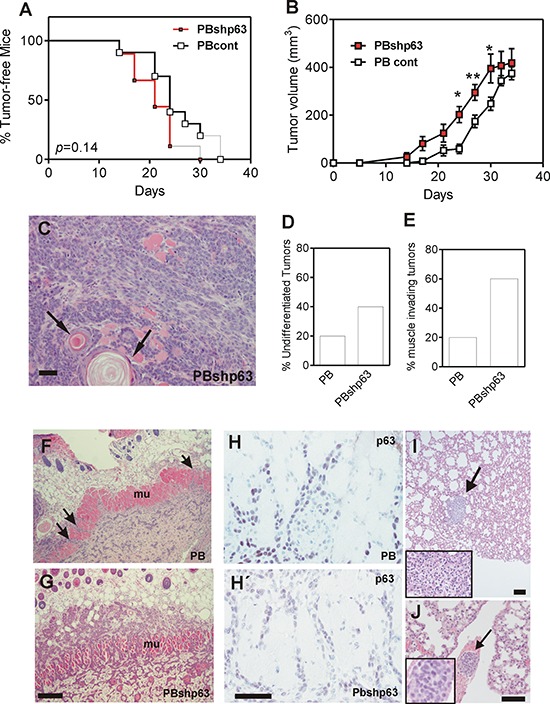
Knock down of p63 promotes tumor dedifferentiation and invasiveness **A.** Tumor incidence upon subcutaneous injection of PB (black line) or PBshp63 cells (red line) determined by Kaplan Meyer curves. *p* value was determined by log rank test. **B.** Tumor growth of subcutaneously injected PB (open squares) or PBshp63 (red squares) cells. *p* Value was determined by 2Way ANOVA followed by Bonferroni post test. *denotes *p* value < 0.05, **denotes *p* value < 0.01. **C.** Representative image of H&E stained subcutaneous tumor produced by the injection of PBshp63 cells showing the presence of undifferentiated and differentiated areas (denoted by arrows). Bar = 250 μm. **D, E.** Summary of differentiation status (D) and subcutaneous muscle invasion (E) of the subcutaneous tumors (*n* = 5 for each group) produced by the injection of PBshp63 cells. **F, G.** Representative images of H&E stained subcutaneous tumor produced by the injection of PB (F) or PBshp63 (G) cells showing the subcutaneous muscle layer invasion (denoted by arrows). Bar = 150 μm. **H, H'.** Representative immunohistochemistry images showing the expression of p63 in the muscle invasive cells of the subcutaneous tumors produced by the injection of PB (H) or PBshp63 (H') cells. Bar = 150 μm. **I.** Metastatic nodule (denoted by arrow) observed upon tumor induction by subcutaneous injection of PBshp63 cells. Inset shows a higher magnification of the metastatic nodule. Bar = 250 μm. **J.** Representative image of H&E stained lung section showing the presence of malignant epithelial cell aggregate (denoted by arrow) in a lung blood vessel. Inset shows a higher magnification of the epithelial cell aggregate. Bar = 150 μm.

We next assayed the *in vivo* tumorigenic properties of control and shp63 PB cells upon subcutaneous injection in *nu/nu* mice. No differences in tumor appearance were observed (Fig. 7A), although tumor growth was slightly faster in p63-silenced cells (Fig. 7B), despite no significant differences in cell cycle profiles were detected between PB and PBshp63 cells (Supp Fig. 2B). The subcutaneous tumors displayed undifferentiated and differentiated areas (denoted by arrows in Fig. 7C). Importantly, tumors produced by PBshp63 cells displayed increased undifferentiated phenotype (Fig. 7D), and increased muscle invasiveness (Fig. 7E, 7F, 7G). Immunohistochemistry analyses revealed that, in parental PB cell-induced tumors, many muscle-invading cells displayed reduced p63 expression (Fig. 7H), similarly to tumors produced by PBshp63 cells (Fig. 7H').

In spite of the increased invasiveness, only one mice subcutaneously injected with PBshp63 cells displayed a small lung nodule (Fig. 7I), whereas none of the controls showed lung metastatic lesions (not shown). We thus monitored the metastatic capacity of control and PBshp63 cells upon intravenous injection. Similarly to the subcutaneously injected mice, no overt metastases were observed one month after the tail vein injection of either control or shp63 cells. Nonetheless, 4 out of 5 mice injected with the p63-silenced cells displayed the presence of epithelial cell aggregates in the lung blood vessels (Fig. 7J). This may indicate that, although the downregulation of p63 favors EMT process, it is insufficient to promote the overt metastatic behavior of the PB keratinocytes due, in part, to an impairment of the extravasation and colonization in the lungs.

### ΔNp63 expression regulates pluripotency genes

**Figure 8:**
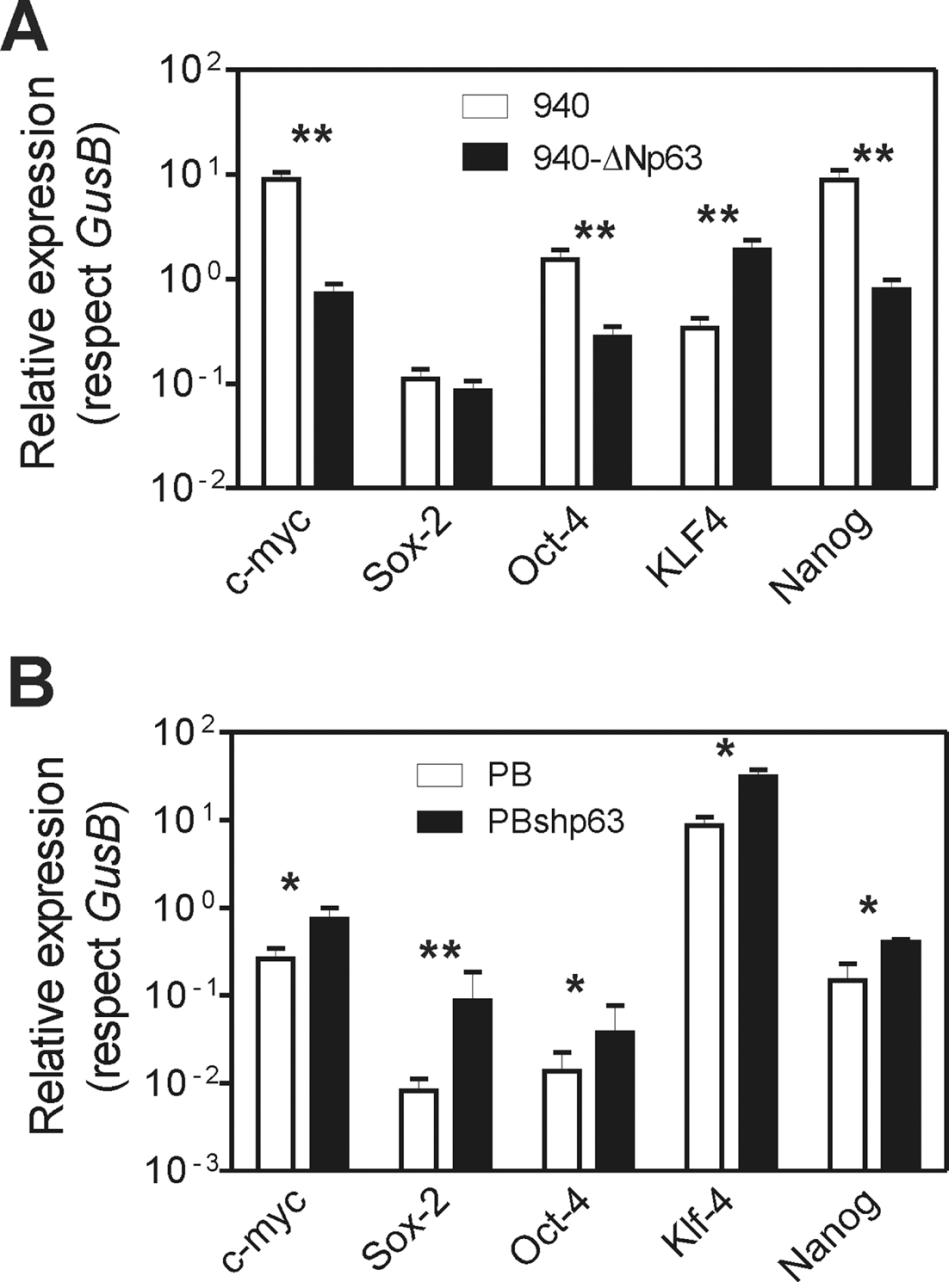
Altered p63 expression promotes altered expression of stemness factors **A, B.** Summary of qRT-PCR determination of the levels of the quoted genes in 940 cells traduced with GFP or ΔNp63α (A), and in PB or PBshp63 cells (B) Data came from at least three independent experiments. *denotes *p* value < 0.05, **denotes *p* value < 0.01 by Mann Whitney *t* Test.

The EMT process is associated with the acquisition of stem cell-like properties [38], and tumors with increased aggressiveness also display ES-like genomic features [36]. Accordingly, cancer cells present some remarkable similarities with embryonic stem cells, including unlimited proliferation and self-renewal, and the expression of pluripotency genes, such as *NANOG*, *OCT4* or *SOX2* [48]. Of relevance, SOX2, OCT4 and NANOG are also particularly involved in squamous cell carcinoma malignization and progression [49–51]. More recently, it has been reported that p63 can also alter the iPS generation from adult epithelial cells [52]. Consequently, the altered metastatic behavior produced by ΔNp63 could also be related to the expression of pluripotency genes. We thus analyzed whether these genes were affected by the forced expression of huΔNp63α in 940 cells, or the reduced expression mediated by shRNA lentiviral constructs in PB keratinocytes. We observed that, in 940 cells (Fig. 8A), the expression of huΔNp63α produced a significant decrease in *Myc*, *Oct4* and *Nanog* genes. In agreement, the reduced expression of p63 was correlated with increased expression of all these genes in PB cells (Fig. 8B). These data revealed that, besides opposing to EMT process, p63 expression may also reduce the stem cell-like properties of p53-deficient mouse keratinocytes, thus probably contributing to the increased malignancy.

## DISCUSSION

The frequent amplification and overexpression of ΔNp63 in multiple epithelial cancers, have led to a general assumption that this protein behaves as an oncogene [12]. In agreement with this, ΔNp63 may repress oncogene-induced senescence [15]. However, it has also been reported that reduction of p63 expression levels favors EMT and metastasis [20, 21, 53], and in aggressive invasive epithelial tumors ΔNp63 is down regulated, suggesting that p63 may play potential roles as metastasis suppressor [16–18]. Here we provide further evidence of this function in the context of p53-deficiency (spontaneous tumors in *Trp53^ΔEpi^* mice and 940 cells) or *Trp53* mutation (PB cells).

The deficiency in p53 confers increased susceptibility to multiple spontaneous tumor development. However, based on mouse KO models [54], it has been assumed that complete loss of function of p53 is not prone to metastasis. In spite of this assumption, the reduced expression of other p53 family members, p63 and p73, produced higher tumor burden and metastatic potential compared to heterozygous deficient p53 mice [26], thus offering the first evidence suggesting that the reduction of p63 confers metastatic potential to transformed p53-deficient cells. Our present data reinforce this finding and provide possible mechanistic explanations for this role of p63 as metastasis modulator.

Tumor metastasis is the major cause of mortality of human cancers. In epithelial tumors, metastasis is associated with the loss of epithelial characteristics and the acquisition of mesenchymal properties by a genetically controlled process named epithelial mesenchymal transition (EMT), which is also essential during embryonic development [55, 56]. These facts have posed tremendous efforts to understand the molecular mechanism controlling EMT in order to find possible therapeutic agents that impair metastatic growth of the tumors. A number of master genes controlling EMT in tumors have been identified [56]. We have previously shown that spontaneous skin tumors caused by the epidermal-specific deficiency in *Trp53* (or simultaneous *Rb1* and *Trp53* [32]) rapidly undergo EMT process and frequently metastatize to the lungs [37]. This process is accompanied by the induction of various EMT-inducing transcription factors and deregulated expression of various miRNAs [37]. Here we show that the transcriptional downregulation of ΔNp63 is an early event during tumor development in the context of p53 deficiency. Moreover, ΔNp63 expression is regained in lung metastasis outgrowths, indicating that the observed downregulation of ΔNp63 is not attributable to gene loss, but rather gene silencing. In addition, the re-expression of ΔNp63 observed in lung metastasis, where the opposite process to EMT, the mesenchymal-epithelial transition, takes place, also suggests that ΔNp63 may play a role in this process. In agreement, the reduced p63 expression in PB keratinocytes produces impaired lung colonization, even when epithelial cell aggregates are commonly observed in the lung blood vessels. However, the absence of p63 expression in the metastatic nodules generated by huΔNp63α-transduced 940 cells argues against this possible strict requirement. Also, it is conceivable that the absence of real metastatic nodules in PBshp63 cells could be related to a distinct pattern of p63 isoforms expressed compared to parental or huΔNp63α-transduced 940 cells, which may interact with different regulators [57], or the intrinsic differences in *Trp53* gene status (deleted in 940 and point mutated in PB cells), which may alter the metastatic behavior [27, 58]. In this regard, it has been demonstrated that TGFβ-induced malignant cell responses are repressed by p63, although the possible specific involvement of p63 isoforms (ΔN or TA) has not be determined, and point mutated *Trp53*, but not p53 silencing, contributes to surpass such inhibition [27]. Consequently, the possible involvement of p63 re-expression in mesenchymal-epithelial transition, and the possible differential involvement of p63 isoforms in the context of mutated or ablated p53, would be the subject of future research.

The potential roles of ΔNp63 preventing EMT are reinforced by the observed downregulation of EMT-mediating transcription factors, such as Snail, Twist, Zeb1, Zeb2 and FoxC2 upon expression of huΔNp63α in 940 cells; whereas p63 silencing in PB cells caused their increased expression (with the exception of Zeb2). Interestingly, the reduced metastatic behavior observed upon expression of huΔNp63α in 940 cells strongly suggests that the elucidation of the possible mechanisms leading to p63 silencing (Supplementary Fig. 1D), could become interesting strategies to prevent metastatic spreading of epithelial cancers characterized by loss of *TP53*.

The ΔNp63 protein is able to directly modulate transcription in a positive and negative manner, and an extremely wide number of transcripts have been found to be under p63 regulation [25, 59]. Indeed, ChIP-Seq approaches have identified the binding of p63 to over 7000 genes [20, 60, 61] and only a subset of these sites are bound by p53 in response to DNA damage [60]. A careful analysis of these ChIP-seq data revealed that p63 does not bind to any of the above mentioned transcription factors mediating EMT [60], thus indicating that the observed deregulation is probably indirect.

Among the factors that are known to modulate the expression of these transcription factors, different miRNAs have emerged as key elements [41, 62]. In this regard, we have also reported that metastatic spreading of p53-deficent epidermal tumors is associated with specific deregulation of various miRNAs [37], and ChIP-seq data identified multiple miRNAs as targets for p63 regulation [20, 60].

In the present study we show that the experimental deregulation of p63 levels promoted altered expression of various miRNA previously involved in EMT and tumor metastasis, including miR-21, miR-34a, miR-200a, miR-200b, miR-203, miR19a and miR205. Of note, several of these miRNAs have been identified in ChIP-seq experiments and display binding sites for p53 (miR-21, miR34a), p63 (miR-200a, miR-200b) or both (miR-205) [20, 60]. Importantly, many of these miRNAs can target various transcription factors modulating EMT and whose expression is affected by p63 levels. Nonetheless, other possible mechanisms cannot be discarded at present. For instance, the altered expression of p63 isoforms can induce a metabolic rewiring in tumor cells [21, 63], and oxidative metabolism could be a critical suppressor of metastasis [64]. Future genomics studies may provide new mechanistic insights about the genes and miRNAs regulated by p63 and the molecular mechanisms underlying the observed functions.

There is wide evidence that the EMT process leads to the acquisition of embryonic stem cell-like features in various human tumors [36, 62, 65, 66]. Moreover, the transcriptome analysis of spontaneous skin tumors of *Trp53^ΔEpi^* mice also revealed a significant overlap with embryonic stem cell like gene signatures [34]. More recently it has been reported that ΔNp63-deficient keratinocytes display increased sensitivity to cell reprogramming to pluripotency by Yamanaka factors [52]. Importantly, most if not all of these factors are also associated with increased malignancy in squamous cell carcinoma [49–51]. We thus investigated whether altered p63 levels could induce changes in transcription factors mediating reprogramming to pluripotency. We found that partial depletion of ΔNp63 is sufficient to induce the expression of *Myc*, *Sox2*, *Oct4* and *Nanog*. Importantly, although none of these factors are directly bound by p63, they display post-transcriptional regulation by various miRNAs identified as targets of p63 [52]. Further, we have recently observed that overexpression of *Nanog* in transgenic mouse skin contributes to EMT by directly modulating the expression of *Zeb2*, *Twist1* and *miR-21* [51].

Collectively, our data reinforce the possible role of p63 as metastasis suppressor in the context of p53 deficiency, through the regulation of various miRNAs and transcription factors that modulate the EMT processes.

## MATERIALS AND METHODS

### Mice and cell lines

Mouse model lacking *Rb1* and *Trp53*, or *Trp53* alone, in the basal layer of the epidermis have been previously described [32]. The 940, COCA and PB keratinocytes have been previously described [37, 42, 46]. To monitor *in vitro* migration and invasion a previously reported protocol was followed [37]. *In vivo* xenografts experiments are detailed in Supplementary Information. All the animal experiments were approved by the Animal Ethical Committee (CEEA) and conducted in compliance with Centro de Investigaciones Energéticas, Medioambientales y Tecnológicas (CIEMAT) guidelines.

### Lentivirus construction

The sequence encoding for the human ΔNp63 gene was obtained by XbaI/XhoI digestion of the ΔNp63α-FLAG vector (Adgene #26979) and inserted into de NheI/XhoI sites of the pL105iGFP backbone lentiviral vector (kindly provided by Dr. R. Murillas). Lentivirus production is detailed in Supplementary Information. For the generation of PBshp63, PB cells were transduced using pLKO.1 Mission Lentiviral system (Sigma, TRCN0000416977 and TRCN0000423330, sequences are provided in Supp Table 2) following manufacturer's recommendations. A control of pLKO.1 lentivirus containing an sh sequence that targets no known mammalian genes was used. Upon infection, clones were selected by puromycin resistance (Sigma, 20 μg/ml) for 2 weeks.

### Quantitative RT-PCR and immunoblotting

Total RNA was isolated from mouse tissue and culture cells and as previously described [37, 67–69]. The qRT-PCR procedures for genes and miRNAs are detailed in Supplementary Materials and Methods and Supplementary Table 1. Total RNA was isolated from mouse tissue and culture cells and as previously described [37, 67–69]. The qRT-PCR procedures for genes and miRNAs are detailed in Supplementary Information. For immunoblots, protein extracts were obtained and processed as described [37]. Detailed protocols and antibodies used are provided in the Supplementary information.

### Immunohistochemistry and immunofluorescence

Samples were fixed in 4% PBS-buffered formalin or 70% ethanol, embedded in paraffin wax and sectioned (5 μm). Cells were grown in glass coverslips and were fixed in cold methanol/acetone for 10 min [70, 71]. Immunohistochemistry and Immunofluorescence were performed using previously described standard protocols [32, 37]. Antibodies used are detailed in Supplementary Information.

## SUPPLEMENTARY METHODS


