# Supplementary material for: The downregulation of ΔNp63 in p53-deficient mouse epidermal tumors favors metastatic behavior
Source: Oncotarget. 2015 Jul 1;6(27):24230–45. doi: 10.18632/oncotarget.4353 (PMC4695182; doi:10.18632/oncotarget.4353)
Supplement: Supplementary file 1 [file oncotarget-06-24230-s001.pdf]

SUPPLEMENTARY METHODS

Xenograft tumor generation

One million of 940 or huΔNp63α-940 cells were delivered via subcutaneous injection in each flank of 10 immunocompromised nude (*nu*/*nu*) mice. Tumor growth was followed every 3 days with external caliper. In half of the mice ($n = 5$ for 940 and $n = 5$ for huΔNp63α-940 cells) tumors were removed by surgery, and the animals were kept alive and were sacrificed one month after surgery and lungs were collected. The remnant mice ($n = 5$ for 940 and $n = 5$ for huΔNp63α-940 cells) were sacrificed and tumors and lungs were collected and preserved in formalin or 70% ethanol. For tail vein injections, cells were grown *in vitro* and directly injected into the circulation of immunodeficient mice (1). In this case, 10^5 cells were injected per mouse. After 5 weeks, mice displayed respiratory distress and were sacrificed due to ethical reasons, and their lungs removed and preserved as previously described. For PB and PBshp63 keratinocytes similar procedures were followed, but all the mice receiving subcutaneous injection were sacrificed ($n = 5$ for PB and $n = 5$ for PBshp63 cells) without surgical procedures.

Lentivirus production

The lentiviral plasmid pL105iGFP ΔNp63α, the pMDLg-pRRE and pRSV-REV packaging vectors, and pMD2-VSVG envelope vector (2) were introduced into 293T cells, using the Polyethylenimine “Max”, (Mw 40,000) system (Polysciences, Inc), and the viral particles generated were used to infect 940 murine cells as described (3). The infected cells were selected by fluorescence-activated cell sorting (FACS). pMDLg-pRRE and pRSV-REV packaging vectors, and pMD2-VSVG envelope vector were kindly provided by Dr. Luigi Naldini (San Raffaele Telethon Institute for Gene Therapy, University Medical School, Milan, Italy).

RNA isolation and PCR procedures

Total RNA was isolated using miRNeasy Mini Kit (Qiagen) according to the manufacturer’s instructions and DNA was eliminated (Rnase-Free Dnase Set, Qiagen). Reverse transcription was performed using the Omniscript RT Kit (Qiagen) and oligo dT primer, using 1 µg of total RNA. PCR was performed in a 7500 Fast Real Time PCR System using Go Taq PCR master mix (Promega) and 1 µl of cDNA as a template. Melting curves were performed to verify specificity and absence of primer dimerization. Reaction efficiency was calculated for each primer combination, and GusB gene was used as reference gene for normalization (4–6). The sequences of the specific oligonucleotides used are listed in Supp Table 1. To measure miRNAs expression quantitatively, RNA was extracted using the same method as above. Reverse transcription was carried out from 10 ng total RNA along with miR-specific primer using the TaqMan® MicroRNA Reverse Transcription Kit (Applied Biosystems). PCR assays were performed using TaqMan® Gene Expression Master Mix and 7500 Fast Real Time PCR System (Applied Biosystems). For normalization, we used RNU6B.

Protein extraction and immunoblot

Whole protein extract from culture cells were lysed by freeze-thawing cycles in lysis buffer (125mM Tris-HCl pH 6.8, 20% glycerol, 4% SDS). Protease (cOmplete Mini EDTA-free, Roche) and phosphatase (PhosSTOP, Roche) inhibitor cocktails were added following manufacturer’s recommendations. 40 µg protein per sample were resolved in 4–20% SDS-PAGE gels (BioRad) and transferred to nitrocellulose membranes (Amersham). Membranes were blocked with 5% non-fat milk diluted in TBS and incubated with the appropriate antibodies diluted in TBS-Tween, 0.5% BSA. Secondary antibodies were purchased from Jackson ImmunoResearch and used at 1/5000. Super Signal West Pico Chemiluminscence Substrate (Pierce) was used according to the manufacturer’s recommendations to visualize the bands. In all cases actin was used for loading control. The antibodies are as follows: Actin (Santa Cruz, diluted 1/500), p63 (mAb 4A4, Santa Cruz, and AbCam diluted 1/500), p53 (NovoCastra, diluted 1/500), Snail (Santa Cruz, diluted 1/500), E-cadherin (Transduction Lab, diluted 1/2500), Twist1 (AbCam, diluted 1/5000), p73 (AbCam diluted 1/200) and ΔNp73α (Imgenex, diluted 1/100).

Antibodies used in immunohistochemistry and immunofluorescence

Primary antibodies against p63 (Abcam, diluted 1/200), E-cadherin (Transduction Lab, diluted 1/50) and keratin K5 (Covance, diluted 1/1000) were used on deparaffinized sections or fixed cultured cells as described previously (7–9). Secondary peroxidase-complexed antibodies, against rabbit IgG (Amersham, diluted 1/5000), mouse IgG (Jackson, diluted 1/5000) and goat IgG (Santa Cruz, diluted 1/10000) were used in immunohistochemistry studies, while Molecular Probes fluorochrome-complexed secondary antibodies

(all diluted 1/1000) were used in Immunofluorescence. Immunofluorescences were counterstained with DAPI (1/100, Roche).

Matrigel invasion chambers

Cells were seeded in BD Biocoat Matrigel Invasion Chambers (BD Biosciences) and 24 hours later, medium and non-invasive cells were removed from the membrane surface. Then, invasive (with matrigel) and migrating (without matrigel) cells were fixed in methanol for 2 minutes and stained with 1% toluidine for 2 more minutes. After 3 washes in water, membranes were set on a slide for microscopic observation. Experiments were performed in quintuplicated and are shown as mean ± SEM.

Cell cycle analysis

For cell cycle analysis detached and adherent cells were collected and incubated in a 4′, 6′-diamidino-2-phenylindole (DAPI) solution (2 μg/ml DAPI, 0.05% NP-40 in PBS 1X). Cell cycle was analyzed using a LSRFortessa (BD Biosciences) flow cytometer and FlowJo software (Tree Star Inc.). Cell debris and aggregated cells were excluded from the analysis. At least 10,000 events were analyzed per sample.

REFERENCES

1. Mohanty S, Xu L. Experimental metastasis assay. J Vis Exp. 2010.
2. Dull T, Zufferey R, Kelly M, Mandel RJ, Nguyen M, Trono D, et al. A third-generation lentivirus vector with a conditional packaging system. J Virol. 1998; 72:8463–71.
3. Barde I, Salmon P, Trono D. Production and titration of lentiviral vectors. Current protocols in neuroscience / editorial board, Jacqueline N Crawley, et al: 2010; Chapter 4:Unit 4 21.
4. Santos M, Martinez-Fernandez M, Duenas M, Garcia-Escudero R, Alfaya B, Villacampa F, et al. *In vivo* disruption of an Rb-E2F-Ezh2 signaling loop causes bladder cancer. Cancer Res. 2014: doi: 10. 1158/0008-5472. CAN-14-1218
5. Saiz-Ladera C, Lara MF, Garin M, Ruiz S, Santos M, Lorz C, et al. p21 suppresses inflammation and tumorigenesis on pRB-deficient stratified epithelia. Oncogene. 2013; doi:10.1038/onc.2013.417.
6. Costa C, Santos M, Martinez-Fernandez M, Duenas M, Lorz C, Garcia-Escudero R, et al. E2F1 loss induces spontaneous tumour development in Rb-deficient epidermis. Oncogene. 2013; 32:2937–51.
7. Bornachea O, Santos M, Martinez-Cruz AB, Garcia-Escudero R, Duenas M, Costa C, et al. EMT and induction of miR-21 mediate metastasis development in Trp53-deficient tumours. Scientific reports. 2012; 2:434.
8. Martinez-Cruz AB, Santos M, Lara MF, Segrelles C, Ruiz S, Moral M, et al. Spontaneous squamous cell carcinoma induced by the somatic inactivation of retinoblastoma and Trp53 tumor suppressors. Cancer Res. 2008; 68:683–92.
9. Paramio JM, Casanova ML, Segrelles C, Mittnacht S, Lane EB, Jorcano JL. Modulation of cell proliferation by cytokeratins K10 and K16. Mol Cell Biol. 1999; 19:3086–94.

Supplementary Table S1: Sequence of primers used in RTqPCR experiments

Name	F sequence	R sequence
ΔNp63	5′-CAAAACCCTGGAAGCAGAAA-3′	5′-GAGGAGCCGTTCTGAATCTG-3′
TAp63	5′-GTGTATGAACCTTCCGAAAA-3′	5′-GAGGAGCCGTTCTGAATCTG-3′
GusB	5′-GAGGATCCACAGTGGCCCA-3′	5′-CAGCCTCCAAGGGGAGGT-3′
Klf-4	5′-AACATGCCCGGACTTACAAA-3′	5′-TTCAAGGGAATCCTGGTCTTC-3′
Sox-2	5′-CCCATGGTGGTGGTACGGGAATTC-3′	5′-TCTCGGTCTCGGACAAAAGT-3′
Oct-4	5′-TAGGTGAGCCGTCTTTCCAC-3′	5′-GCTTAGCCAGGTTCGAGGAT-3′
c-myc	5′-TAACTCGAGGAGGAGCTGGA-3′	5′-GGCCTTTTCATTGTTTTCCA-3′
Nanog	5′-GCCTCCAGCAGATGCAAG-3′	5′-GGTTTTGAAACCAGGTCTTAACC-3′
Twist1	5′-AGCTACGCCTTCTCCGTC-3′	5′-TCCTTCTCTGGGAAACAAT-3′
FoxC2	5′-GCTTTCCTGCTCATTCGTCTT-3′	5′-AAATATCTTACAGGTGAGAGGCAA-3′
Zeb1	5′-AGGTGATCCAGCCAAACG-3′	5′-GGTGGCGTGGAGTCAGAG-3′
Zeb2	5′-AAGCCAGGGACAGATCAGC-3′	5′-CACACTCCGTGCACTTGA-3′
Snai1	5′-CACCTCCAGACCCACTCAGAT-3′	5′-CCTGAGTGGGGTGGGAGCTTC-3′
E-cad	5′-ATCCTCGCCCTGCTGATT-3′	5′-ACCACCGTTCTCCTCCGT-3′

Supplementary Table S2: Sequences of shp63 lentiviral constructs

5′ CCGGTGATCGATGCCGTGCGCTTTACTCGAGTAAAGCGCACGGCATCGATCATTTTTTG-3′
5′. CCGGGAATGAACAGACGTCCAATTTCTCGAGAAATTGGACGTCTGTTCATTCTTTTTTG.3′

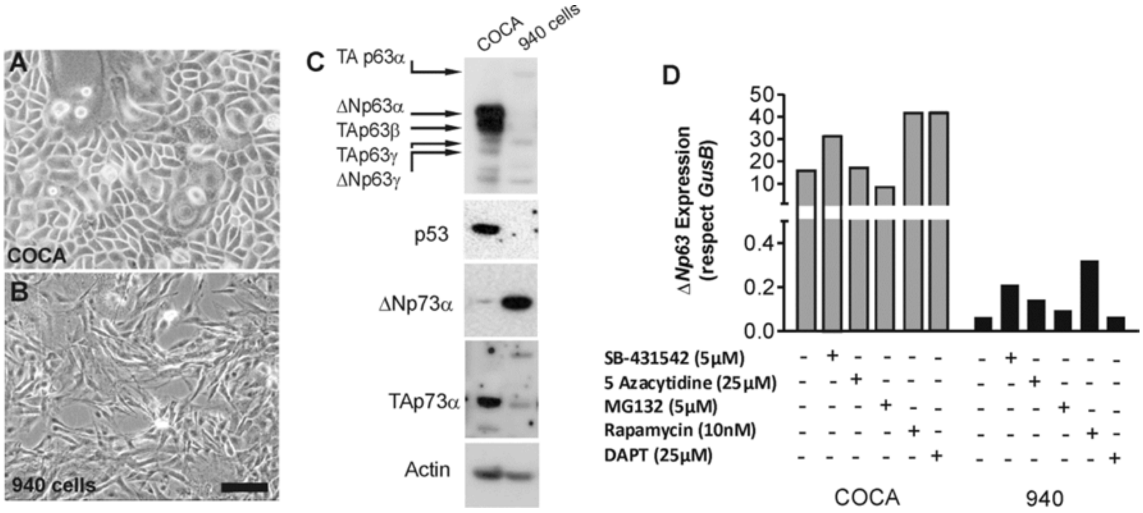

Supplementary Figure S1: Expression of p53 family members in 940 cells. **A–B.** Representative examples of 940 (A) and COCA (B) keratinocyte morphology as assessed by phase contrast of cell cultures. Bar = 150 µm **C.** Immunoblots showing the expression of the quoted proteins in extracts from COCA and 940 keratinocytes. **D.** RTqPCR analysis of the expression of ΔNp63 gene in COCA and in 940 cells treated for 24 h with the quoted pharmacological inhibitors at the stated concentrations.

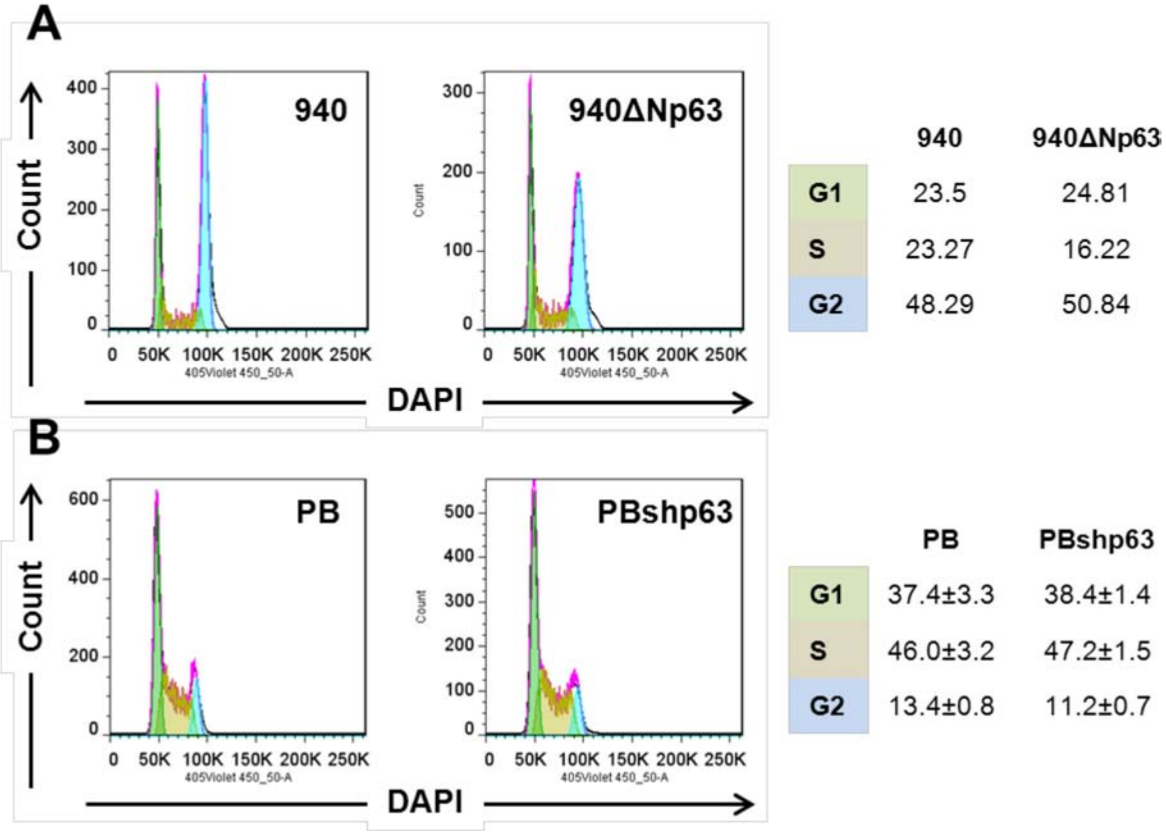

Supplementary Figure S2: Cell Cycle analyses. A. Cell cycle analysis using FlowJo software of 940 control cells or transduced with huΔNp63α (940ΔNp63) **B.** Cell cycle analysis using FlowJo software of PB control keratinocytes or infected with the lentiviral construct shp63.
